# Cervical Cytological Abnormalities in Women with Inflammatory Bowel Disease (IBD) and Non-IBD Controls: A Retrospective Study

**DOI:** 10.3390/jcm15218324

**Published:** 2026-10-26

**Authors:** Edyta Maria Tulewicz-Marti, Hanna Szweda, Patrycja Gumula, Izabela Sierakowska, Konrad Lewandowski, Tadeusz Issat, Grazyna Rydzewska

**Affiliations:** 1 Department of Gastroenterology and Internal Medicine, National Medical Institute of the Ministry of the Interior and Administration, Woloska 137 St., 02-507 Warsaw, Poland; 2 Department of Obstetrics and Gynaecology, Institute of Mother and Child in Warsaw, 01-211 Warsaw, Poland; 3 IBD Clinical and Research Center, ISCARE a.s., 190 00 Prague, Czech Republic; 4 Collegium Medicum, Jan Kochanowski University, 25-317 Kielce, Poland

**Keywords:** IBD, cervical cytology, Crohn’s disease, ulcerative colitis, Bethesda System, human papillomavirus

## Abstract

**Background:** Data on cervical cytological abnormalities in women with inflammatory bowel disease (IBD) are inconsistent. We compared their prevalence in women with and without IBD. **Methods:** In this retrospective single-centre analysis of electronic medical records (2022–2024), women with Crohn’s disease (CD) or ulcerative colitis (UC) were compared with 141 women treated at a gynaecology outpatient clinic. Cytology was classified exclusively using the Bethesda System (abnormal = ASC-US or worse; “Pap II” inflammation = normal). **Results:** Of 301 women with IBD, 280 (141 CD, 139 UC) had evaluable cytology. The most recent cytology assessment was abnormal in 4/280 women with IBD (1.4%) versus 13/141 controls (9.2%; OR 0.14, 95% CI 0.05–0.45); a current or historical abnormality was identified in 12/280 women with IBD (4.3%) versus 13/141 controls (9.2%; OR 0.44, 95% CI 0.20–0.99; *p* = 0.051). CD and UC did not differ, and no clinical or treatment variable was associated with abnormality (12 events). hrHPV testing was documented in 15.7% of women with IBD (controls 85.8%). **Conclusions:** Abnormal cytology was infrequent in this closely monitored IBD cohort and numerically lower than in controls; however, the controls comprised a selected group, HPV testing differed between groups, and events were few, so an increased risk cannot be excluded. Regular gynaecological surveillance remains essential.

## 1. Introduction

Inflammatory bowel disease (IBD) is a chronic inflammatory condition of the gastrointestinal tract encompassing ulcerative colitis (UC) and Crohn’s disease (CD). In recent years, the incidence of IBD has increased globally, including in Asian countries; as a result, new medications have been formulated and introduced [1]. Evidence regarding the association between IBD and cervical intraepithelial neoplasia (CIN) remains inconclusive; however, women with IBD, particularly those who are immunocompromised or smoke, may be at increased risk for CIN [2,3,4]. Therefore, regular screening and preventive measures are recommended for this population [5]. Additionally, some studies indicate that immunocompromised women, such as those with lupus, may be more susceptible to HPV infection and at greater risk of abnormal Pap smears [6]. With the introduction of new and innovative drugs, observations regarding their influence on women’s health are needed. 

The primary aim of this study was to retrospectively assess the prevalence of and risk factors for abnormal cervical cytology, classified according to the Bethesda System, in women with IBD, considering variables such as disease type, gynaecological history, and both past and current medications. The secondary objective was to evaluate the risk of abnormal cytology in women with IBD compared to women without IBD and to describe the available information on HPV testing and HPV vaccination.

## 2. Materials and Methods

### 2.1. Study Population

In this retrospective study, the demographic data and clinical history of women with IBD, based on electronic medical records from 2022 to 2024, were analysed. The cytology results of women with IBD followed at the Department of Gastroenterology and Internal Medicine, National Medical Institute of the Ministry of the Interior and Administration, Warsaw, were evaluated. The following variables were examined: age, age at IBD diagnosis, disease duration, smoking status, disease phenotype, current and past medication use, gynaecological assessment, and current and past cytological abnormalities. Gynaecological history and medication use, including current and past immunosuppressive, small-molecule drugs, and biological treatments, were assessed. The data were compared with those of women without IBD (controls) who underwent cervical screening at the Department of Obstetrics and Gynaecology, Institute of Mother and Child, Warsaw, Poland.

### 2.2. Inclusion and Exclusion Criteria

The study period extended from January 2022 to December 2024. Patients were eligible for inclusion if they were female; aged ≥18 years; had a confirmed diagnosis of CD or UC based on standard clinical, endoscopic, histological, and radiological criteria; and had at least one cervical cytology result recorded in their electronic medical record during the study period. Women were excluded from the cytological analyses if they had undergone total hysterectomy (*n* = 12 identified after extraction of the database, including two women who had been treated for cervical cancer), had a pre-existing diagnosis of cervical cancer or high-grade cervical intraepithelial neoplasia prior to IBD diagnosis, or had incomplete gynaecological or medication records precluding classification. The 12 women who underwent hysterectomy are described in the tables providing demographic and gynaecological history below (*n* = 301).

### 2.3. Control Group Selection

Control patients were women without a diagnosis of IBD who underwent routine cervical cancer screening at the Department of Obstetrics and Gynaecology, Institute of Mother and Child, Warsaw, during the same study period; they were recruited from the gynaecology department of a separate institution and are referred to as women without IBD, not as representatives of the general population. Controls were selected at random from among the women routinely examined at the gynaecology outpatient clinic and were matched to the IBD group for age. The controls were required to have no history of chronic inflammatory, autoimmune, or immunosuppressive therapy-requiring disease and at least one recorded cytology result during the study period.

### 2.4. Definition and Classification of Cervical Cytology

Cervical smears (conventional or liquid-based) were reported and analysed exclusively according to the 2014 Bethesda System for reporting cervical cytology [7]; the older Papanicolaou classification (classes I–V) was not applied for analysis. In Poland, cervical cytology has historically been reported according to the Papanicolaou classification, which assigns smears to one of five groups reflecting the presumed risk of cervical carcinoma: group I (normal cytology), group II (benign inflammatory or reactive changes), group III (unclear or suspicious findings, corresponding to mild or moderate dysplasia and often subdivided into IIIA and IIIB), group IV (severe dysplasia or carcinoma in situ) and group V (invasive cancer). This system has been replaced in current practice and in the national screening programme by the Bethesda System for Reporting Cervical Cytology (TBS), which describes the cellular findings directly and is now the international standard. Because Papanicolaou groups are not one-to-one equivalent to Bethesda categories, all results in the present study are reported and analysed exclusively according to the Bethesda System (2014) [7]. Where historical records used Papanicolaou groups, these were translated to the approximate Bethesda correlates shown in Table 1; in particular, groups I and II (inflammation and reactive changes) were classified as negative for intraepithelial lesions or malignancy (NILM), i.e., as normal, and were not counted as cytological abnormalities.

Abnormal results (epithelial cell abnormalities) comprised atypical squamous cells of undetermined significance (ASC-US); atypical squamous cells, cannot exclude HSIL (ASC-H); low-grade squamous intraepithelial lesion (LSIL); high-grade squamous intraepithelial lesion (HSIL); atypical glandular cells (AGC); and squamous cell carcinoma. Any result of ASC-US or worse was considered a cytological abnormality [7]. Abnormal results were also categorized as low-grade (ASC-US, LSIL/CIN1) and high-grade or clinically significant (ASC-H, HSIL/CIN2+, AGC, and squamous cell carcinoma). Histological or excisional findings, gynaecological history, hrHPV test results, and HPV vaccination status were extracted from the medical records where documented.

A “historical” cytological abnormality was defined as any abnormality of ASC-US or worse (or histologically confirmed CIN or excisional treatment for CIN/HSIL) recorded in the patient’s electronic medical record prior to the most recent cytology assessment, including results obtained before the start of biologic therapy, while a “current” abnormality referred to the most recent recorded result between January 2022 and December 2024. A “cumulative” abnormality (denoted “current or historical” in the tables) was defined as an abnormal result in either of these two categories. The absence of a documented prior abnormality does not necessarily indicate a confirmed normal cytological history, as it may instead reflect an undocumented or unperformed test.

Gynaecological history was extracted as free text and grouped into obstetric history (pregnancy, delivery or caesarean section, in vitro fertilisation, ectopic pregnancy), benign uterine or adnexal conditions (fibroids, ovarian cysts, endometriosis, polycystic ovary syndrome, polyps, endometrial hyperplasia), hysterectomy, menstrual disorders or menopause, previous cervical excision (conisation), and other conditions (e.g., vaginal or perianal fistula, infection); categories are not mutually exclusive. Results of high-risk HPV (hrHPV) testing were recorded as positive or negative, and HPV vaccination as vaccinated, not vaccinated, or information only. Because the nationwide HPV vaccination programme in Poland has only been implemented since 1 June 2023 [8], most women in this cohort (with a mean age of about 40 years) were not eligible for programme vaccination, and hrHPV testing was not part of routine primary screening during the study period; both HPV-related variables were therefore documented only for a minority of women, were not systematically collected, and are reported descriptively. Vaccination data were not available for the control group.

Cytological examinations were performed in two laboratories, each located in the centre from which the participants were recruited (the gastroenterology centre for women with IBD and the gynaecology department for controls), using the same method and reported according to the same Bethesda classification in both groups. hrHPV testing, when performed, was based on the routine clinical assay performed in the respective centre.

### 2.5. Missing Data

Women with missing cytology results or incomplete data on key demographic, clinical, or pharmacological variables were excluded from the relevant analysis on a variable-by-variable (complete-case) basis; no imputation methods were applied.

**Figure 1:**
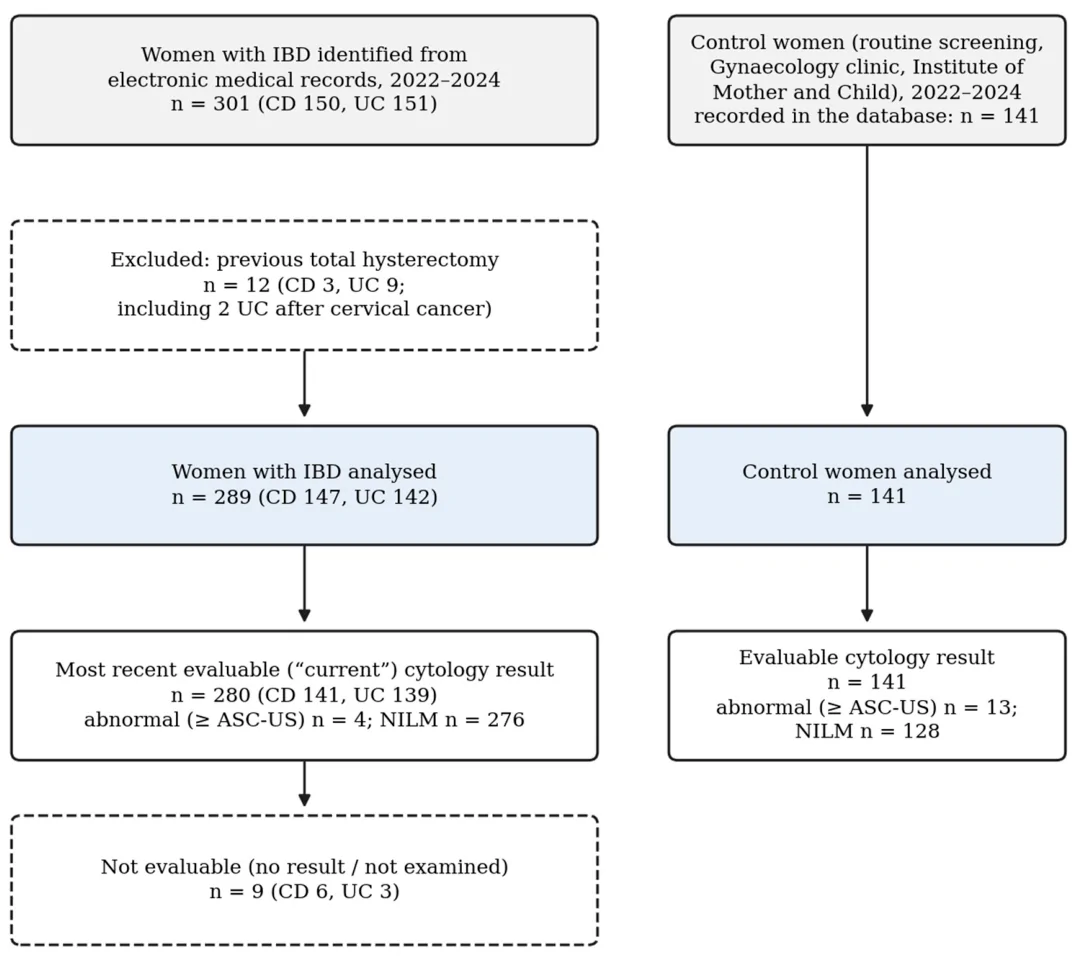
Flow diagram of study participants. CD—Crohn’s disease; UC—ulcerative colitis.

Of the 301 women with IBD, 12 (3 CD, 9 UC) had undergone hysterectomy and were excluded from the cytological analyses, leaving 289 women (147 CD, 142 UC). For 9 of them (6 CD, 3 UC), no evaluable cytology result was available (no result or never examined), so that 280 women (141 CD, 139 UC) formed the evaluable population for the Bethesda-based analyses (Figure 1); the corresponding denominators are reported alongside each percentage in the Results section (see Section 3). Information on gynaecological history, hrHPV testing, and HPV vaccination was incomplete (see Section 2.4). Women with missing data on other variables were excluded from the relevant analysis on a variable-by-variable (complete-case) basis. Analysis included only known data; missing data were not imputed.

The flow of participants is shown in Figure 1 (STROBE-style flow diagram). To assess the potential impact of missing data, the 9 women with non-evaluable cytology were compared with the 280 evaluable women with IBD. The groups did not differ significantly in age (34.2 ± 19.1 vs. 39.6 ± 13.0 years, *p* = 0.229), proportion of participants with UC (33.3% vs. 49.6%, *p* = 0.501), smoking status (*p* = 0.481), current immunosuppressive therapy (*p* > 0.999) or current biologic/small-molecule therapy (*p* = 0.217); with only nine women, however, the power of this comparison was very limited.

### 2.6. Definition of Medication Exposure

Current medication use was defined as active treatment with the specified immunosuppressive, small-molecule, or biologic agent at the time of the most recent cervical cytology assessment. Past medication use was defined as any prior exposure to the specified agent before the current assessment, irrespective of whether treatment had since been discontinued.

Because current medication use was defined at the time of the most recent cytology assessment, while the cytological outcome (current or historical) could reflect an abnormality recorded at any earlier point in the patient’s history, the temporal sequence between treatment initiation and the occurrence of cytological abnormality was not established. Consequently, associations reported between specific therapies and cytological abnormality reflect the co-occurrence of exposure and outcome rather than a confirmed causal or temporal relationship; a cumulative-exposure or time-to-event analysis would be required to address causality directly.

### 2.7. Primary and Secondary Endpoints

The primary endpoint was the prevalence of abnormal cervical cytology according to the Bethesda System (ASC-US or worse), defined as the current (most recent) result and as the cumulative (current or historical) abnormality, in women with IBD and in controls. Secondary endpoints included the comparison of CD and UC; the comparison according to the type of immunosuppressive therapy (AZA vs. MP/MTX); the exploratory assessment of clinical, phenotypic, pharmacological and gynaecological factors associated with cumulative abnormality; and the descriptive reporting of inflammatory/reactive changes (NILM), hrHPV testing and HPV vaccination.

### 2.8. Statistical Analysis

Statistical analyses were conducted using R version 4.2.1 (R Core Team (2022). R: Language and environment for statistical computing by the R Foundation for Statistical Computing, Vienna, Austria) for the original descriptive analyses and Python 3.11 (pandas 3.0, SciPy 1.17; Firth penalised regression implemented by the authors) for the Bethesda-based re-analysis. Nominal variables are presented as the number of observations and the percentage of the group, while continuous variables are reported as the mean ± standard deviation (SD) or median with interquartile range (Q_1_–Q_3_), depending on the distribution. Normality was assessed using the Shapiro–Wilk test and visual inspection of histograms. Group comparisons were performed using the chi-square test or Fisher’s exact test for nominal variables and the *t*-test or Mann–Whitney U test for continuous variables, as appropriate. Mean or median differences (MDs) and odds ratios (ORs) with a 95% confidence interval (CI) were calculated. All statistical tests were two-tailed, with α = 0.05.

Absolute differences in prevalence are reported with Wald 95% confidence intervals. Comparisons of the frequency of abnormal cytology were performed using Fisher’s exact test; when a cell was empty, the odds ratio and its confidence interval were calculated after adding 0.5 to each cell (Haldane–Anscombe correction). Because only 12 cumulative (4 current) abnormalities were observed, regression analyses were considered exploratory. Associations were estimated using univariable Firth penalised-likelihood logistic regression (one predictor per model) to limit small-sample bias and separation, and no multivariable model with more than the minimum number of covariates was fitted (events per variable < 10). The comparison of women with IBD and controls was additionally adjusted for age and smoking using Firth regression. In sensitivity analyses, the nine non-evaluable women were counted as either all normal or all abnormal (extreme-case scenarios). No formal equivalence or non-inferiority analysis was performed; non-significant results are therefore not interpreted as evidence of equivalent risk.

### 2.9. Study Size and Potential Sources of Bias

All eligible women were included (convenience sample), and no a priori sample size or power calculation was performed; the precision of the estimates is therefore described using 95% confidence intervals. Several measures were taken to limit bias. To reduce selection bias, all eligible women with IBD identified from the electronic medical records were included, and controls were selected at random from routinely screened women and matched for age. To limit misclassification of the outcome, all results were classified according to the Bethesda System; benign inflammatory or reactive changes (formerly Pap II) were classified as normal, and cytology was performed using the same method in both centres. Differences in the use of hrHPV testing between groups (and its selective use in women with abnormal cytology), potential differences in screening frequency, completeness of medical records, and the gynaecological setting of the controls could not be eliminated and are acknowledged as limitations. hrHPV results are reported descriptively only, and the comparison between the groups is interpreted cautiously. Missing data were handled by complete-case analysis, and the effect of missing cytology results was examined in sensitivity analyses. Confounding by age and smoking was addressed by matching and adjustment, but residual confounding (e.g., by screening history) cannot be excluded.

## 3. Results

Data were collected from 301 women with IBD (150 with CD [49.8%] and 151 with UC [50.2%]) and 141 control women. Twelve women with IBD (3 CD, 9 UC) had undergone hysterectomy (two after cervical cancer) and were excluded from the cytological analyses, leaving 289 women, of whom 280 (141 CD, 139 UC) had an evaluable cytology result (Figure 1). The mean age in the IBD group was 36.96 ± 11.92 years for patients with CD and 43.19 ± 14.42 years for patients with UC (mean difference = −6.23, 95% CI [−9.23; −3.23]), while the control group had a mean age of 41.01 ± 12.66 years. No significant difference in disease duration was identified between the groups. Additionally, no significant differences were found regarding smoking status, current or past exposure to immunosuppressive drugs, or frequency of gynaecological examinations. Current use of advanced therapies (biologic or small-molecule agents) was reported in 88.0% of women with CD and 74.2% of women with UC (OR = 2.55, 95% CI [1.36; 5.01], *p* = 0.003). In total, 30.7% of women with CD and 31.1% of women with UC had been treated with advanced therapies in the past (*p* > 0.999). According to the Bethesda System, the most recent (current) cytology was abnormal (ASC-US or worse) in 2 of 141 evaluable women with CD (1.4%) and 2 of 139 evaluable women with UC (1.4%) (OR = 0.99, 95% CI [0.14; 7.10], *p* > 0.999). Current or historical (cumulative) abnormalities were noted in 7 of 141 evaluable women with CD (5.0%) and 5 of 139 evaluable women with UC (3.6%) (OR = 1.40, 95% CI [0.43; 4.52], *p* = 0.770). Inflammatory or reactive changes without epithelial abnormality (NILM; formerly “Pap II”) were recorded for 6 women with CD (4.3%) and 18 with UC (12.9%) (OR = 0.30, 95% CI [0.11; 0.78], *p* = 0.010) and were not counted as abnormal. Table 2 presents these data, as well as the phenotype characteristics of women with CD according to the Montreal classification.

No significant association was observed between abnormal cervical cytology and the type of immunosuppressive therapy administered (AZA vs. MP/MTX), but the numbers were extremely small. Among evaluable women, the frequency of current abnormalities was 0/10 (0.0%) in women currently receiving MP/MTX and 1/74 (1.4%) in those receiving AZA (OR = 2.33 after correction, 95% CI [0.09; 61.11], *p* > 0.999). Cumulative abnormalities were found in 1/10 (10.0%) and 5/74 (6.8%), respectively (OR = 1.53, 95% CI [0.16; 14.64], *p* = 0.544; see Table 3).

An additional analysis compared the evaluable women with IBD (*n* = 280) with the control group (*n* = 141). The mean age was 39.63 ± 13.02 years in the IBD group and 41.01 ± 12.66 years in the controls. No significant difference in age or smoking was observed between the groups (*p* = 0.300 and *p* > 0.999, respectively; see Table 4).

Current abnormal cytology according to the Bethesda System (ASC-US or worse) was found in 4 of 280 evaluable women with IBD (1.4%) versus 13 of 141 control women (9.2%) (OR = 0.14, 95% CI [0.05; 0.45], *p* = 0.0003); the absolute difference was −7.8 percentage points (95% CI −12.8 to −2.8). Current or historical (cumulative) abnormalities were found in 12 of 280 women with IBD (4.3%) versus 13 of 141 controls (9.2%) (OR = 0.44, 95% CI [0.20; 0.99], *p* = 0.051; absolute difference −4.9 percentage points, 95% CI −10.3 to +0.4).

After adjustment for age and smoking (Firth penalised regression, *n* = 416), the odds of current abnormality remained lower in women with IBD (OR = 0.14, 95% CI [0.05; 0.42], *p* < 0.001), as did the odds of cumulative abnormality (OR = 0.41, 95% CI [0.18; 0.92], *p* = 0.031). Extreme-case sensitivity analyses for the 9 non-evaluable women (n = 289), assuming that all had normal cytology, yielded current 4/289 (1.4%) vs. 13/141, *p* = 0.0002, and cumulative 12/289 (4.2%) vs. 13/141, *p* = 0.047; assuming that all were abnormal yielded current 13/289 (4.5%) vs. 13/141, *p* = 0.082, and cumulative 21/289 (7.3%) vs. 13/141, *p* = 0.568. These associations must not be interpreted as a protective effect of IBD or its treatment: the control group consisted of women attending a gynaecology department, and hrHPV testing was performed far more often in controls (85.8%) than in women with IBD (15.7%) (see the Discussion).

In univariable Firth regression (Table 5), none of the demographic, disease-related or treatment variables was significantly associated with cumulative abnormal cytology (*p* > 0.10 for all). A recorded gynaecological history was associated with higher odds of cumulative abnormality (7/83 [8.4%] vs. 5/197 [2.5%]; OR = 3.40, 95% CI [1.09; 10.58], *p* = 0.035), but this was entirely explained by previous conisation for CIN/HSIL being counted as gynaecological history: after excluding the women whose only gynaecological entry was a cervical excision, the frequencies were 3/79 (3.8%) vs. 9/201 (4.5%) (*p* > 0.999). No multivariable model of risk factors was fitted because of the small number of events (12).

Montreal classification variables were not modelled because several categories contained no or very few events (separation), which had previously produced unreliable estimates (e.g., OR > 1000 or <0.001). With only 12 events, all estimates are imprecise and should be regarded as exploratory; the positive association of any gynaecological history with cumulative abnormality is explained by previous conisation (see text). The model for gynaecological history includes 278 women because this information was missing in two women, who are counted as having no history in the descriptive comparison in the text.

The distribution of results by Bethesda category and the histological or excisional findings are shown in Table 6. In the most recent cytology, all four current abnormalities in women with IBD were ASC-US (*n* = 3) or HSIL (*n* = 1; treated by conisation in 2023); no LSIL, ASC-H, AGC or carcinoma was recorded. A further eight women had a historical abnormality (CIN1/LSIL *n* = 4, CIN2 *n* = 1, HSIL *n* = 1, dysplasia of unspecified grade *n* = 1, ASC-US *n* = 1) with a currently normal cytology. Four women had undergone conisation. Twenty-four women (8.6%) had only inflammatory/reactive changes or “Pap II”, which are NILM. hrHPV test results were available for only 44 of 280 evaluable women with IBD (15.7%; 24 CD, 20 UC), of which 8 (18.2%) were positive. HPV vaccination status was documented for 68 women (24.3%), and 52 of them (76.5%) were vaccinated; 6 further women had only received information about vaccination. Because the nationwide HPV vaccination programme in Poland only began on 1 June 2023 [8], these data are incomplete and are therefore reported descriptively. Individual data for all 12 women with a current or historical abnormality, including HPV results and vaccination status, are reported in Table 7.

Gynaecological history was recorded for 97 of the 301 women with IBD (32.2%) and did not differ between CD and UC groups (Table 8). The most frequent entries were benign uterine or adnexal conditions (fibroids, ovarian cysts, endometriosis) and obstetric history. Benign uterine or adnexal conditions were recorded more often in patients with CD than in those with UC (20.0% vs. 10.6%, *p* = 0.025); this exploratory observation was not corrected for multiple comparisons. Twelve women (4.0%) had undergone hysterectomy, two (both with UC) for cervical cancer, and four had a previous cervical excision (conisation).

## 4. Discussion

In this retrospective study of 301 women with IBD, cytological abnormalities classified strictly according to the Bethesda System were uncommon: the most recent cytology assessment was abnormal (ASC-US or worse) in 4 of 280 evaluable women (1.4%), and a current or historical abnormality was identified in 12 of 280 (4.3%). Both figures were numerically lower than in the control group (9.2%; OR = 0.14, 95% CI [0.05; 0.45] for current and OR = 0.44, 95% CI [0.20; 0.99], *p* = 0.051, for cumulative abnormality). This comparison must be interpreted with caution. The controls were women attending a gynaecology outpatient clinic, for whom abnormal results are expected to be enriched (due to referral for symptoms or previous abnormal smears), whereas the women with IBD were examined during gastroenterological follow-up; hrHPV testing was performed in 85.8% of controls but only 15.7% of women with IBD, indicating different ascertainment and screening histories, which may differ [9], could not be compared. Due to these differences and the small number of events, our data do not allow conclusions about the relative risk of cervical abnormality in IBD. Instead, they describe a low frequency of abnormalities in a closely monitored cohort.

The literature is inconsistent on this subject. Some studies reported an increased risk of cervical abnormalities for women with IBD, particularly for those exposed to immunosuppressive therapy [4,10,11,12], whereas others found no association [13]. The low prevalence of current abnormality in our cohort does not refute these observations; differences between studies may reflect the populations examined (referral centre versus population-based cohorts), screening intensity, the definition of abnormality (Bethesda categories versus the older Papanicolaou classes; pooling of low- and high-grade lesions), HPV prevalence and vaccination coverage, and statistical power. Our data cannot exclude a modest-to-moderate increase in risk compared with the general population, and population-based meta-analyses report a higher incidence of low-grade cervical lesions in IBD, although not a significant increase in cervical cancer [12]. The low frequency of abnormalities in our cohort may be related to the very high rate of regular gynaecological follow-up (99.3% in the CD group and 96.6% in UC). Further, the contribution of modern therapies, with which 74–88% of women included in this study are being treated, cannot be assessed in this design.

A methodological point deserves emphasis: cervical cytology should be reported exclusively according to the Bethesda System [7]. The Papanicolaou class system used in older records is obsolete; its classes do not correspond one-to-one to Bethesda categories, and class II (“inflammation”, “reactive changes”) is NILM under the Bethesda classification. In our database, 24 evaluable women (8.6%; 6 CD, 18 UC) had only such changes. Counting them as abnormal, as some were categorized in the source database, would have inflated the apparent prevalence several-fold and even changed the direction of the comparison with controls, in whom only epithelial abnormalities were recorded. Inflammatory changes were more frequent in UC patients than in those with CD (12.9% vs. 4.3%, *p* = 0.010); we have no explanation for this finding, which may be due to chance, genital infections, topical therapy or variable reporting of benign findings.

Abnormalities were equally rare in the CD and UC groups, both in the most recent cytology (2/141 [1.4%] vs. 2/139 [1.4%]; OR = 0.99, 95% CI [0.14; 7.10], *p* > 0.999) and cumulatively (7/141 [5.0%] vs. 5/139 [3.6%]; OR = 1.40, 95% CI [0.43; 4.52], *p* = 0.770), although the number of events is too small to detect modest differences. Women with CD were younger than those with UC (36.96 ± 11.92 vs. 43.19 ± 14.42 years; *p* < 0.001) and received advanced therapy more often (88.0% vs. 74.2%; OR = 2.55, 95% CI [1.36; 5.01], *p* = 0.003). Neither the type of immunosuppressive therapy (cumulative abnormality in 1/10 women on MP/MTX and 5/74 on AZA; OR = 1.53, 95% CI [0.16; 14.64], *p* = 0.544) nor any other clinical, phenotypic, or pharmacological variable was significantly associated with abnormal cytology in univariable Firth regression; however, with 12 events, the confidence intervals were wide (e.g., OR = 3.42, 95% CI 0.77–15.24 for smoking, a recognised risk factor [13]), and no conclusion on a higher or lower risk with any agent can be drawn. ECCO guidelines point to a potentially increased risk of high-grade cervical dysplasia in women receiving thiopurines, and the AGA recommends age-appropriate cervical cancer screening for all women with IBD [14,15,16]; adherence to cervical screening is therefore strongly advised.

Gynaecological history was recorded in about one-third of women and comprised mostly benign uterine or adnexal conditions and obstetric history; the higher frequency of benign uterine or adnexal conditions among the women with CD (20.0% vs. 10.6%) is an exploratory finding. The association of any gynaecological history with cumulative abnormality was entirely due to previous conisation, itself a consequence of an abnormal cytology result, and disappeared when these women were excluded. Twelve women (4.0%) had undergone hysterectomy, two of whom had UC and were treated for cervical cancer; these clinically important cases were excluded from the cytological analyses and cannot be interpreted without information on the timing of diagnosis in relation to IBD onset, immunosuppression, and screening.

HPV information was available for only a minority of women. hrHPV testing was documented in 15.7% of evaluable women with IBD (8 of 44 tested were positive) and probably performed selectively in women with abnormal cytology, so these proportions cannot be interpreted as prevalence or compared with controls (85.8% tested; 9.1% positive). HPV vaccination was documented for 24.3% of women with IBD, and 76.5% of those documented were vaccinated. Because the nationwide HPV vaccination programme in Poland was only launched on 1 June 2023 [8], most women in this age range were not eligible for vaccination under this programme, and vaccinations recorded in their medical files were probably individual and selected. Therefore, no conclusion on the effect of vaccination can be drawn. HPV testing and vaccination should be recorded prospectively in IBD cohorts.

This study has several limitations. First, its retrospective single-centre design and low event rate (4 current and 12 cumulative abnormalities among 280 women) resulted in wide confidence intervals and limited power; regression analyses are exploratory, Montreal variables could not be modelled, and no multivariable model of risk factors was fitted (apart from the age- and smoking-adjusted comparison with controls). Second, free-text and Papanicolaou-type entries were translated into Bethesda categories retrospectively, which may involve misclassification, and because all non-NILM results were analysed together, low-grade abnormalities could not be separated from clinically significant lesions; colposcopy and histology were documented only occasionally. Third, hrHPV and vaccination data were incomplete and selective. Fourth, because medication exposure was defined at the time of the most recent cytology, whereas the outcome could include earlier abnormalities, the temporal sequence could not be established; the completeness of cytology records may have differed between the groups, and the absence of a documented prior abnormality does not confirm a normal history. Fifth, gynaecological history was categorised retrospectively from free text, twelve women who underwent hysterectomy were excluded, and nine had no evaluable cytology (without significant differences from the evaluable women but with very limited power). Finally, the controls were selected women attending a gynaecology clinic of a different institution with differential ascertainment (hrHPV testing: 85.8% vs. 15.7%), which limits comparability and generalisability. Our results should therefore be regarded as hypothesis-generating.

## 5. Conclusions

Abnormal cervical cytology was infrequent in this closely monitored cohort of women with IBD (current 1.4%, cumulative 4.3%) and numerically lower than in the control group, though this comparison must be interpreted cautiously. Inflammatory or reactive changes (formerly Pap II) are NILM and must not be counted as abnormal. Because the controls comprised a selected group, HPV testing differed markedly between groups, and the number of events was small, the results do not allow for conclusions about relative risk and cannot exclude a clinically meaningful increased risk compared with the general population. Considering the risks associated with immunosuppression noted in the ECCO and AGA guidelines [14,15,16], routine gynaecological monitoring and adherence to cervical screening remain essential. Future prospective studies with larger cohorts, extended follow-up, Bethesda-based reporting, and standardised hrHPV co-testing and documentation of HPV vaccination are warranted to refine risk stratification and optimise screening protocols for women with IBD.

## Figures and Tables

**Table 1:** Papanicolaou groups used in Poland and their approximate correlation with the Bethesda System.

Papanicolaou Group (Poland)	Cytological Description	Approximate Bethesda Category	Classified in This Study as
I	Normal cytology	NILM	Normal
II	Inflammatory, reactive or atrophic changes, no epithelial abnormality	NILM (reactive or other non-neoplastic findings)	Normal
III (IIIA/IIIB)	Unclear findings; mild to moderate dysplasia	ASC-US, ASC-H, LSIL (CIN1)	Abnormal (low-grade or unclear)
IV	Severe dysplasia, carcinoma in situ	HSIL (CIN2/3)	Abnormal (high-grade)
V	Invasive carcinoma	Squamous cell carcinoma	Abnormal (high-grade)

NILM—negative for intraepithelial lesion or malignancy; ASC-US—atypical squamous cells of undetermined significance; ASC-H—atypical squamous cells, cannot exclude HSIL; LSIL/HSIL—low-/high-grade squamous intraepithelial lesion; CIN—cervical intraepithelial neoplasia. The correlation is approximate and shown for orientation only; the classification used for analysis was Bethesda.

**Table 2:** Comparative analysis of ulcerative colitis (UC) and Crohn’s disease (CD) groups.

	UC		CD		MD/OR (95% CI)
	*n*	*n* = 151	*n*	*n* = 150	
Age [years], mean ± SD	151	43.19 ± 14.42	150	36.96 ± 11.92	−6.23 (−9.23;−3.23)
Age at IBD diagnosis [years], mean ± SD	150	32.14 ± 14.24	149	26.32 ± 11.60	−5.82 (−8.78;−2.87)
Disease duration [years], median (Q_1_; Q_3_)	150	10.00 (5.00;14.00)	149	9.00 (5.00;15.00)	−1.00 (−2.00;1.00)
Smoking status, *n* (%)					
Current smoker	151	7 (4.6)	150	11 (7.3)	-
Former smoker		1 (0.7)		1 (0.7)	
Never smoked		143 (94.7)		138 (92.0)	
Phenotype, *n* (%)					
Montreal A					
N/A			149	1 (0.7)	
A1				15 (10.1)	
A2				117 (78.5)	
A3				16 (10.7)	
Montreal L					
L1			150	5 (3.3)	
L2				31 (20.7)	
L3				107 (71.3)	
L4				1 (0.7)	
N/A				6 (4.0)	
Montreal B					
B1			150	73 (48.7)	
B2				30 (20.0)	
B3				34 (22.7)	
N/A				13 (8.7)	
Medication exposure, *n* (%)					
Current use of immunosuppressive therapy	151	43 (28.5)	150	45 (30.0)	1.08 (0.64;1.82)
AZA		37 (24.5)		40 (26.7)	1.12 (0.65;1.95)
MP/MTX		7 (4.6)		5 (3.3)	0.71 (0.17;2.67)
Previous use of immunosuppressive therapy	151	30 (19.9)	150	25 (16.7)	0.81 (0.43;1.51)
AZA		24 (15.9)		21 (14.0)	0.86 (0.43;1.71)
MP/MTX		1 (0.7)		1 (0.7)	1.01 (0.01;79.50)
Biologic agents or small-molecule therapies					
Current use	151	112 (74.2)	150	132 (88.0)	2.55 (1.36;5.01)
Previous use		47 (31.1)		46 (30.7)	0.98 (0.58;1.64)
Gynaecological assessment, *n* (%)	146	141 (96.6)	138	137 (99.3)	4.86 (0.56;42.12)
Current abnormal cytology (Bethesda ≥ ASC-US), *n* (%)	139	2 (1.4)	141	2 (1.4)	0.99 (0.14;7.10)
Cumulative abnormal cytology (current or historical), *n* (%)		5 (3.6)		7 (5.0)	1.40 (0.43;4.52)
Inflammatory/reactive changes or Pap II (NILM), *n* (%)		18 (12.9)		6 (4.3)	0.30 (0.11;0.78)
Any gynaecological history, *n* (%)	151	47 (31.1)	150	50 (33.3)	1.11 (0.68;1.79)

Groups were compared using the chi-square test or Fisher’s exact test for nominal variables and the *t*-test or Mann–Whitney U test for continuous variables, as appropriate. CI—confidence interval; MD—mean/median difference for continuous variables, calculated as women with CD minus women with UC. OR—odds ratios for nominal variables, calculated as women with CD versus women with UC. Demographic, smoking, phenotype, treatment and gynaecological history rows refer to all 301 women identified; cytology rows refer to the 280 evaluable women after exclusion of hysterectomy (denominators shown). Abnormal cytology was defined according to the Bethesda System (ASC-US or worse); inflammatory/reactive changes and “Pap II” were classified as NILM. Odds ratios with an empty cell were calculated with the Haldane–Anscombe correction.

**Table 3:** Deviation from normative values vs. the type of immunosuppressive therapy administered.

	MP/MTX	AZA	OR (95% CI)	*p*
Current abnormal cervical cytology (Bethesda ≥ ASC-US), *n* (%)	0 (0.0) of 10	1 (1.4) of 74	2.33 (0.09;61.11) *	>0.999
Current or historical abnormal cervical cytology, *n* (%)	1 (10.0) of 10	5 (6.8) of 74	1.53 (0.16;14.64)	0.544

Data are presented as the number of women (percentage of group in column). Groups were compared using Fisher’s exact test. CI—confidence interval, OR—odds ratios calculated for MP/MTX women compared to AZA women. * Haldane–Anscombe correction (0.5 added to each cell) because of an empty cell.

**Table 4:** Patient characteristics.

	IBD		Non-IBD		*p*
	*n*	*n* = 280	*n*	*n* = 141	
Age [years], mean ± SD	280	39.63 ± 13.02	141	41.01 ± 12.66	0.300
Current or former smoker, *n* (%)	280	19 (6.8)	136	9 (6.6)	>0.999
Comorbidities (any), *n* (%)	n/a	n/a	137	14 (10.2)	n/a
Alcohol use, *n* (%)	n/a	n/a	136	4 (2.9)	n/a

Groups were compared using the chi-square test for smoking status and the *t*-test for age. Comorbidities and alcohol use were recorded for controls only (n/a—not available in a comparable form for women with IBD, in whom other diseases were recorded as free text); no formal comparison was made.

**Table 5:** Univariable Firth penalised logistic regression for cumulative abnormal cytology (Bethesda ≥ ASC-US, current or historical; 12 events).

Predictor Variable	*n*	OR	95% CI	*p*
Ulcerative colitis (vs. Crohn’s disease)	280	0.73	0.24 to 2.27	0.591
Age [per year]	280	0.98	0.94 to 1.03	0.485
Age at IBD diagnosis [per year]	279	0.98	0.94 to 1.03	0.496
Disease duration [per year]	278	1.00	0.93 to 1.08	0.930
Current or former smoker	280	3.42	0.77 to 15.24	0.106
Current immunosuppressive therapy	280	2.43	0.79 to 7.46	0.122
Current AZA therapy	280	2.11	0.67 to 6.58	0.200
Current MP/MTX therapy	280	3.56	0.53 to 23.83	0.190
Previous immunosuppressive therapy	280	0.56	0.10 to 3.23	0.520
Current biologic or small-molecule therapy	280	1.73	0.30 to 9.90	0.539
Previous biologic or small-molecule therapy	280	1.78	0.57 to 5.54	0.320
Any gynaecological history (incl. previous conisation)	278	3.40	1.09 to 10.58	0.035

CI—confidence interval, OR—odds ratio, AZA—azathioprine, MP—mercaptopurine, MTX—methotrexate. Each row is a separate model (one predictor); *n* varies with missing data. OR for UC is relative to CD; the other ORs refer to the presence of the characteristic (or to a one-year increase).

**Table 6:** Cervical cytology according to the Bethesda System, histological/excisional findings, hrHPV testing, and HPV vaccination in evaluable women with IBD and in controls.

Category (*n* of Women)	IBD (*n* = 280)	CD (*n* = 141)	UC (*n* = 139)	Controls (*n* = 141)
Most recent (current) cytology, Bethesda				
NILM	276 (98.6)	139 (98.6)	137 (98.6)	128 (90.8)
of which inflammatory/reactive change or “Pap II”	24 (8.6)	6 (4.3)	18 (12.9)	n/a
ASC-US	3	1	2	9
LSIL	0	0	0	2
HSIL	1	1	0	1
ASC-H/AGC/squamous cell carcinoma	0	0	0	1 (ASC-H)
Any abnormality (≥ASC-US)	4 (1.4)	2 (1.4)	2 (1.4)	13 (9.2)
Earlier (historical) abnormality, current result NILM				
ASC-US	1	0	1	n/a
CIN1/LSIL	4	3	1	n/a
CIN2	1	1	0	n/a
HSIL (treated by conisation)	1	0	1	n/a
Dysplasia, grade not specified	1	1	0	n/a
Any historical abnormality	8 (2.9)	5 (3.5)	3 (2.2)	n/a
Cumulative abnormality (current or historical)	12 (4.3)	7 (5.0)	5 (3.6)	13 (9.2)
Excision/conisation documented	4	3	1	n/a
hrHPV test result available	44 (15.7)	24 (17.0)	20 (14.4)	121 (85.8)
hrHPV positive (of those tested)	8 (18.2)	6 (25.0)	2 (10.0)	11 (9.1)
HPV vaccination status documented	68 (24.3)	35 (24.8)	33 (23.7)	not recorded
Vaccinated against HPV (of those documented)	52 (76.5)	29 (82.9)	23 (69.7)	not recorded

Data are *n* (%); percentages in the “Any historical abnormality” row use the group denominators. Bethesda 2014 categories: NILM—negative for intraepithelial lesion or malignancy; ASC-US—atypical squamous cells of undetermined significance; ASC-H—atypical squamous cells, cannot exclude HSIL; LSIL/HSIL—low-/high-grade squamous intraepithelial lesion; AGC—atypical glandular cells; CIN—cervical intraepithelial neoplasia. Inflammatory/reactive changes and “Pap II” are NILM and are not abnormalities. Two women with UC who had undergone hysterectomy for cervical cancer were excluded from the table (see Figure 1). n/a—not available (for controls, only abnormal results were recorded; vaccination status was not recorded).

**Table 7:** Individual data of the 12 women with IBD and a current or historical cytological abnormality (ASC-US or worse).

No.	Disease	Age Band (Years)	Current Cytology (Bethesda)	Historical Abnormality	Conisation	hrHPV Test	HPV Vaccination
1	UC	40–49	ASC-US	–	No	n.d.	Not vaccinated
2	CD	≥50	NILM	Dysplasia, grade not specified	No	Positive	Vaccinated
3	CD	40–49	NILM	CIN1/LSIL	Yes	Positive	Vaccinated
4	UC	30–39	NILM	HSIL	Yes	n.d.	n.d.
5	UC	<30	NILM	CIN1/LSIL	No	n.d.	n.d.
6	CD	40–49	NILM	CIN2	No	n.d.	Vaccinated
7	CD	30–39	NILM	CIN1/LSIL	Yes	Positive	Vaccinated
8	UC	<30	ASC-US	–	No	n.d.	n.d.
9	CD	<30	HSIL	–	Yes	n.d.	n.d.
10	UC	<30	NILM	ASC-US	No	Positive	Vaccinated
11	CD	30–39	ASC-US	–	No	n.d.	n.d.
12	CD	30–39	NILM	CIN1/LSIL	No	Negative	Vaccinated

Age is given in bands. n.d.—not documented (hrHPV test not performed or result not recorded; vaccination status not documented). hrHPV was tested in 5 of the 12 women (4 positive) and vaccination status was documented in 7 (6 vaccinated). Historical abnormality refers to earlier results with a currently normal cytology; “–” means none. Conisation—cervical excision documented. CD—Crohn’s disease; UC—ulcerative colitis; NILM—negative for intraepithelial lesion or malignancy; ASC-US—atypical squamous cells of undetermined significance; HSIL—high-grade squamous intraepithelial lesion; LSIL—low-grade squamous intraepithelial lesion; CIN—cervical intraepithelial neoplasia.

**Table 8:** Gynaecological history in women with IBD (all 301 identified women; categories are not mutually exclusive).

Gynaecological History	IBD (*n* = 301)	CD (*n* = 150)	UC (*n* = 151)	*p*
Any gynaecological history recorded:	97 (32.2)	50 (33.3)	47 (31.1)	0.712
Obstetric history (pregnancy, delivery/caesarean section, IVF, ectopic pregnancy)	24 (8.0)	10 (6.7)	14 (9.3)	0.524
Menstrual disorders/menopause	8 (2.7)	4 (2.7)	4 (2.6)	>0.999
Previous conisation for CIN/HSIL	4 (1.3)	3 (2.0)	1 (0.7)	0.371
Hysterectomy (any indication)	12 (4.0)	3 (2.0)	9 (6.0)	0.138
performed for cervical cancer	2 (0.7)	0 (0.0)	2 (1.3)	0.498
Other (vaginal/perianal fistula, infection, urinary incontinence, other)	11 (3.7)	8 (5.3)	3 (2.0)	0.138

Data are *n* (%). Fisher’s exact test for CD vs. UC. IVF—in vitro fertilisation; CIN—cervical intraepithelial neoplasia; HSIL—high-grade squamous intraepithelial lesion.

## Data Availability

The data presented in this study are available on request from the corresponding author. The data are not publicly available due to privacy and ethical restrictions.

## Abbreviations

The following abbreviations are used in this manuscript:

IBD	Inflammatory Bowel Disease
CD	Crohn’s Disease
UC	Ulcerative Colitis
CIN	Cervical Intraepithelial Neoplasia
HPV	Human Papillomavirus
hrHPV	high-risk Human Papillomavirus
AZA	Azathioprine
MP	Mercaptopurine
MTX	Methotrexate
OR	Odds Ratio
CI	Confidence Interval
SD	Standard Deviation
MD	Mean/Median Difference
ECCO	European Crohn’s and Colitis Organisation
AGA	American Gastroenterological Association
NILM	Negative for Intraepithelial Lesion or Malignancy
ASC-US	Atypical Squamous Cells of Undetermined Significance
ASC-H	Atypical Squamous Cells, cannot exclude HSIL
LSIL	Low-grade Squamous Intraepithelial Lesion
HSIL	High-grade Squamous Intraepithelial Lesion
AGC	Atypical Glandular Cells
